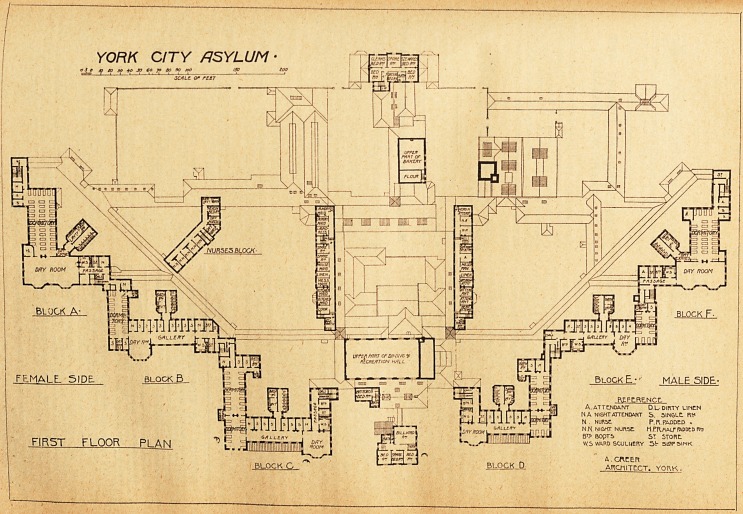# York City Asylum

**Published:** 1907-01-12

**Authors:** 


					YORK CITY ASYLUM.
This asylum is built at Naburn, near York, and was
opened during the year 1906. It contains 362 beds, but the
administrative departments are large enough for about 486
beds. The aspect of the main buildings is south-east, which
is a very good one for an asylum of this design. From the
plans herewith published it will be seen that it is designed
on the principle introduced to the asylum world more than
a generation since by Dr. Asa Gray, of Buffalo; but most,
or perhaps all, of the English architects inspired by Gray
have cut off the apex of the broad arrow, thus obtaining
a rather better frontage. By far the best example of this
plan with which we are acquainted is the New Gloucester-
shire Asylum, in which the blocks are kept distinct; hence
there is a better chance of free circulation of air. At York
the blocks are contiguous, and thus is lost one great advan-
tage of the block plan. As the asylum is a small one, and
never likely to be a very large one, we think the architect
would have done better by studying some of the older plans;
bringing them up to date, rather than adopt this, at present,
fashionable plan, because, as carried out, next to nothing
is gained in point of separation; much is lost in point of
supervision.
The centre block to the south contains rooms for the
assistant medical officers. North of this small block are
the dispensary, office, and pathological room; and to the left
on entering, or west, but approached from the main corridor,
is the matron's room. All these rooms and offices are con-
veniently placed, but we failed to notice any office for the
matron, in the absence of which she must keep her books
and see the nurses in her sitting-room. Further north than
the main south corridor is the dining- and recreation-room,
with a large stage and dressing-rooms at one end. This
room is intended to serve a double purpose which in practice
is never found satisfactory and could hardly be necessary
in an asylum which cost the large sum of ?360 per bed.
Further north than this room is the kitchen department,
placed within corridors arranged almost as a square, having
at the extreme end the general store-room. The component
sections of this department are well placed in relation to
each other and to the blocks. West of the enclosing corridor,
running north and south, are the mess-rooms and sitting-
r?oms for the female staff; and east of the other corridor are
the corresponding rooms for the male staff. The female and
the male visiting-rooms adjoin these respectively, and are
approached from the main corridor and are within reason-
able distance of the main entrance to the asylum. That
entrance block occupies its best position to the extreme
North. It contains committee-room, medical superinten-
dent's office, assistant medical officer's office, chaplain's
l00m, clerk's office, waiting-room and luncheon-room. We
did not see any room for admitting patients in, unless it be
lntended to use the waiting-room for this purpose.
Returning to the main south corridor we enter a passage at
right angles to the corridor, and here is placed the main
staircase to the block. Going southwards along the passage
(female side) we find the boot-room, store-room, and the
ward scullery, and then enter the day-room. This room
is well lighted and has some cross-ventilation. It opens
into a gallery having five windows and two glazed doors.
In front is a verandah, and at the back are six single-bedded
rooms, two nurses' rooms, and the entrance to the sanitary
block, the latter being thus placed in an enclosed court and
having some of the dormitory windows within 20 feet of it.
The sanitary block is necessarily a two-story one; and it
must obstruct much of the morning sun from the dormitorv.
The dormitory is approached from the gallery. It contains
twenty-seven beds. The dimensions are not marked on the
plan before us; but it would seem to be about 85 feet long
and 23 feet wide, and with a 12 feet ceiling would give
about 876 cubic feet of air per bed, an amount which is
certainly small for sick patients. There is a fair amount of
cross-ventilation in the dormitory, as only about one-third
of the east wall is blocked; but then it is divided into two
sections by a partition which to some extent must interfere
with the free circulation of air. At the north end of the
dormitory are bath-room, closet, two single-bedded rooms,
and a nurses' room. Close to the latter is a passage leading
to the staircase and to the main corridor running north-west.
Here the design has one good point. It provides a staircase-
at each end of each block. There is no dining-room attached
to this ward; and, as it is the infirm ward, most of the-
patients not actually bedridden must have their meals in
the day-room, an arrangement which is quite indefensible.
Others of the infirm class will, perhaps, be sent to the
general dining-hall, a practice which is still worse than
giving them their meals in the day-room. The six single-
bedded rooms, which are to be occupied by sick patients
possioly for weeks or months at a time, have a northern
instead of a southern aspect ; and surely this is a great pity.
The first floor of this block is similar to the ground floor,
except that there is no verandah.
The next block, westwards, provides accommodation for
sixty recent and acute cases, thirty on each floor. The plan
is a good deal like that of the first block. There are eight
single-bedded rooms placed to the north on each floor, and
only two on each floor are to the south, while space to the-
south for three others is wasted on a store-room and a boot-
room. The gallery part of the ward has only five windows,
all on the south side. The end of the day-room is formed
by a large bay containing seven windows. Other windows:
it has none. One side is blocked by the dormitory; nearly
all the other side by the gallery and the north end by the
single-bedded rooms. Beyond the bay window theie is.
not even an attempt at cross-ventilation, unless the doors
leading to the dormitory and gallery be kept open or hav e?
open fanlights. The dormitory itself is fairly good as.
272 THE HOSPITAL. Jan. 12. 1907.
GROUND FLOOR PLAN L. J-^ ROOM ? | gW !
e 3 ej I Wrw r^m ~ F <=?!
Ef;4 JPI' J?sf ^Jr S^?er
^^-JlUU ?I*?[Ni 4il| l$|f| fej }?*?? l  kn.L J
? ^ ^j 1
?-ww^- ^?4 &"?? j v A. CREER
BLOCK G- ??11-":- BLOCK D ARCHITECT. YORK
A MO'? BLOCK
A ATTENDANT . D.L OIRTY LlHEN
N A MlfefAT ATTE.NDAMT S SIN&UL R?
M N'JRSE P.R PAODC.D ?
N.N.NKbnT NURSE RPR.HAU*BU>DED R?
B1? BOOTS ST STORt
WS WARD SCULLERY 5k S12P"5INK
GROUND FLOOR PLAN
Jan. 12, 1907, THE HOSPITAL. 273
YORK CITY ASYLUM
C Sj> fij ?0 50 ?+& JO 60 JO GO 90 /CO
SCALL or r?BT
MALE SIDE-
fiEFC-RENCC.
A. ATTENDANT D L- DIRTY LINCN
N A NISHT ATTENDANT S. 3IN&LC.RM
N. NURSE. P.fVPADDED ?
N N NlGrtT NURSC. M.PR.MAIT PADDED R9
BT? BOpTh 5T STORE.
FIRST FLOOR PLAN ^ JBS. ? 1 ~ld ?5 ward scumcry 5- s^k
A.GREER
BLOCK G ' "       BLOGKJJ ARCHITECT. YORK .
FIRST FLOOR PI AN
274 THE HOSPITAL. Jan. 12, 1907,
regards this point of cross-ventilation. Although this ward
is intended for recent and acute cases, it, too, is destitute of
a dining-room.
The adjoining block is for the epileptics and chronic
patients. It has room for eighty patients, forty on each
floor. On leaving the main corridor the ward is reached by
a passage?apparently about 30 feet long. On one side of
the passage are the staircase and boot-room, and on the other
side are the nurses' room, scullery, and store-rooms; and
we do not see how the passage obtains any direct light; but
it may have sufficient borrowed light. The day-room is a
fine room. It has to the south two large bay windows and
on the west side are three windows; but one end is blocked
by the adjuncts already named, and one side by the end of
the dormitory and by a single-bedded room. The latter is.
however, very useful in an epileptic ward. The dormitory
has one end placed against the day-room, and the other is
blocked by the single-bedded wards; but it has five windows
on one side arid four on the other, and there must be a fair
amount of cross-ventilation, but the two beds nearest the
day-room should be removed. The dormitory is about
57 feet long by 27 feet wide, and is probably about 12 feet
high, and has 52 beds. Allowing a margin of error in
measuring a small scale drawing, each patient would have
about fifty superficial feet of floor space and about 600 cubic
feet of air space. Of course these patients are out of bed
by day, but even then 600 feet is not much, especially when,
as in this case, the beds are placed three in a row across the
dormitory. This ward, like the others, has no dining-room.
Where are the epileptics to have their meals ?
The night nurses' rooms are placed in one of the courts
with the object of securing more quietude for them when
in bed by day.
The laundry is placed" to the north. It contains every-
thing essential to a first-rate laundry?general washing-
room, ironing-room, drying-room, foul-linen washhouse and
drying-room, and there is also an officers' laundry.
Each side of the asylum is provided with a general bath-
room. The power-house and the workshops occupy a site on
the male side corresponding to that of the laundry on the
female side.
The medical superintendent's residence is detached from
the asylum. This is a most desirable arrangement and the
architect is to be congratulated on seeing it. The chapel is
also detached from the main building, but we doubt whether
this is a good plan. It is more expensive to build and to
keep up, entails more work on the part of the staff, and fewer
patients are able to use it.
Electric light is used throughout. For the dining hall
the plenum system of warming has been used; but other
parts are warmed by open fireplaces and by steam coils.
All the pipes are carried in suitable channels placed under
the corridor floors, and workmen are enabled to reach them
from the outside. The sewage is connected with the Cor-
poration works, and the water is provided by the York
Water Company.
The architect was Mr. A. Creer, of York, and the con-
tractors for the buildings were Messrs. Longden and Son.
The cost is given as ?130,000, but it is not stated whether
or not this included the site.

				

## Figures and Tables

**Figure f1:**
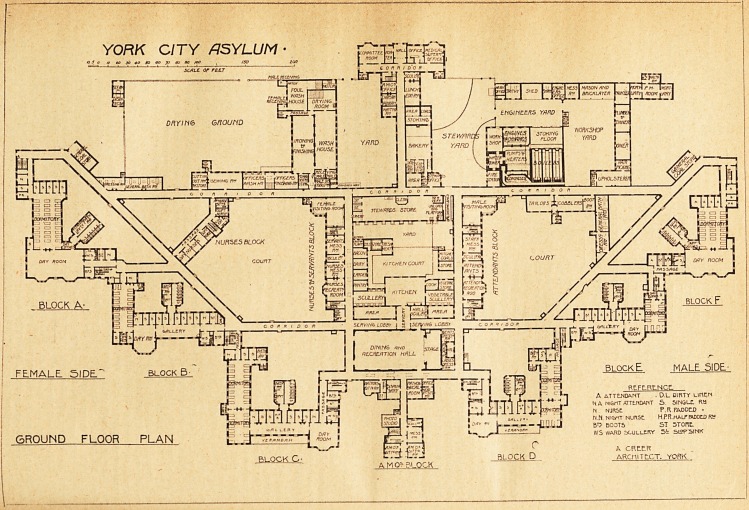


**Figure f2:**